# Reshaping Australian drug policy: the dilemmas of generic medicines policy

**DOI:** 10.1186/1743-8462-4-11

**Published:** 2007-06-01

**Authors:** Hans Löfgren

**Affiliations:** 1 School of International and Political Studies, Deakin University, Melbourne, Australia

## 

With this edition, *Australia New Zealand Health Policy *publishes four articles on the theme of Australian and international generic medicines market dynamics and policy dilemmas. Changes soon to be introduced to pricing arrangements under Australia's Pharmaceutical Benefits Scheme (PBS) make this focus particularly topical. Beecroft presents a community pharmacy perspective on these issues. Faunce and Lexchin explore the vexed issue of 'evergreening' in Australia and Canada, with a comparative emphasis on the implications of bilateral trade agreements between these countries and the US. In a second article, Faunce investigates the generics sector from an industrial renewal perspective, arguing that it would be perilous to fail to develop a systematic approach to the promotion of this industry grounded in public good considerations. The present author provides an analysis of international generics markets which highlights the rise of competitive Indian suppliers.

Most medicines do not have to be expensive. Manufacturing costs are generally low and many off-patent drugs can be sourced at prices which reflect intense competition in global markets. In September 2006 in Florida, for example, Wal-Mart announced that each of 331 generic drugs, covering 143 compounds in more than twenty therapeutic categories, would be made available for a monthly cost of US$4.00. This scheme has since been extended across the US and competitors have introduced similar programs [1]. In Australia and elsewhere, governments are introducing changes to drug policy in order to extend the use of cheaper generic medicines. They face two central questions: to what extent and in what ways should the pricing of patented medicines be linked to the pricing of out-of-patent drugs, and how can the cost of complex and expensive sales and distribution systems be contained?

PBS pricing arrangements have, until now, largely precluded Australian tax payers from benefiting from the ready availability of cheap generics. To address this anomaly, the Health Minister, Tony Abbott, announced a policy package in November 2006 intended to ensure 'better value' through lower prices in the off-patent market [2]. As the number of major drugs facing patent expiry multiplied, pressures for lower generics prices had become increasingly compelling. The proposed reforms are intended to rein in the interrelated problems of unnecessarily high PBS prices for generics and the large discounts (labelled 'secret kickbacks' in the media) provided to pharmacists by generics suppliers. It leaves to one side more difficult questions of transparency and accountability in the pricing of 'innovative' brand name pharmaceuticals.

The government is hoping that the new initiatives will create a stable medicines policy environment for years to come. This follows a period of uncertainty which peaked with the stop-gap introduction in 2005 of a mandatory 12.5% price cut on the launch of the first alternative brand of an already PBS-listed drug. Following furious brand name industry lobbying the government stepped back from mandating, as initially intended, a repeat price cut on the introduction of each subsequent brand [3].

As the number of big-selling out-of-patent drugs increases, so does the possibility of achieving large PBS cost savings through lower generics prices. But the realisation of this potential requires a new pricing model acceptable to all the major stakeholders: the manufacturers of patented drugs and Australia's small group of specialised generics suppliers, pharmacists, wholesalers, prescribers and consumers. Through a lengthy period of behind-the-scenes discussions, the government appears to have come up with a reform package which enjoys the support of major interest groups, most importantly the suppliers of originator brands (through Medicines Australia) and the Pharmacy Guild of Australia. This required the ditching of models for tendering of generics, and for mandatory prescription by generic name, though these had possibly served as government bargaining chips rather than serious proposals.

The off-patent drug market in Australia is already large and growing, comprising more than half of all PBS items. Around 40% of listings have generic competitors and a further 12%, though still patented, are affected through the reference pricing system (explained briefly below) [4]. In value terms, patent-expired PBS drugs represent annual sales of about $3 billion, with generics (non-originator brands) accounting for about $1.3 billion or 43%. This gives non-originator brands a share of total PBS expenditure of around 18%, or about 25–30% of prescriptions [5]. Alphapharm, the dominant generics company in Australia, is the leading supplier to the PBS in volume terms, ahead of Pfizer [6]. But in comparison with the UK and the US, where well above 50% of prescriptions are dispensed with generics, and European countries such as the Netherlands (around 44%) and Denmark (around 70%), the proportion of prescriptions dispensed with a generic remains small in Australia. Originator products retain the lion's share of the off-patent market and, in many cases, continue to be the only brand available, particularly where its price is relatively low, with consequent weak commercial incentives for entry by new suppliers. Originator brands continue to do well, even where a generic alternative is available: in this market segment, almost 40% of prescriptions are still dispensed with a brand premium.

The dominance of brand suppliers is explained by typically small PBS price differentials between originator and generics brands. Pricing incentives are consequently weak for doctors and consumers to choose a generic rather than the originator brand. The PBS listing and pricing process has hitherto delivered relatively favourable prices for patented drugs but, as noted by the Pharmaceutical Benefits Pricing Authority (PBPA), 'in general, the prices Australian taxpayers pay for generic medicines are high compared to some other OECD countries' [[6], p. 12]. The reference pricing system, administered by the PBPA, is at the centre of this conundrum. It ensures that price cuts flow through to all other suppliers of the same or similar products, and there is thus no incentive for suppliers to compete on price to the PBS. Generics suppliers have instead found an inroad into the PBS market though discounts or large 'trading terms' offered to pharmacists, commonly around 30% and sometimes in the order of 50% or more. In other words, pharmacists have been reimbursed by the government at prices well above the prices actually paid. From a pharmacy perspective, these 'trading terms' are considered ordinary business deals which reward efficiencies and scale, and have come to be integrated into overall profitability, as explained by Beecroft. These discounts have also provided incentives for pharmacists to encourage the uptake of generics by consumers. In their absence, the more attractive option would be support for the brand names rather than investing effort in converting patients to generics. But the fact that a large share of the cost benefits of cheap generics has flowed to pharmacists through these discounts, while PBS generics prices continued to approximate those of the originator brands, has riled the government.

Reference pricing, a basic premise of the PBS system, is, as already noted, intended to deliver similar prices for drugs with identical or similar therapeutic effects. Most OECD countries apply some form of reference pricing, though this approach is almost always accompanied by heated controversy [7,8]. In Australia, prices of alternative brands and drugs in the same therapeutic class are key factors taken account of by the PBPA when considering pricing issues. The critical question is: precisely which products should be considered interchangeable and, on that basis, be assigned the same (reimbursement) price? It is accepted almost universally that, to all intents and purposes, alternative generic brands are identical to originator products. The contrary position used to be pressed strongly by originator companies but cannot be held credibly today, more than twenty years after the 1984 US Waxman-Hatch Act, which created the modern generics industry. But where are the lines to be drawn where products have similar therapeutic effects without being chemically identical? Should patented products be referenced against off-patent drugs which deliver similar therapeutic effects? Here opposed perspectives clash: innovator companies seek premium pricing for all newly patented drugs, irrespective of the extent of their marginal benefits compared with previously available alternatives. In contrast, the mandate of the PBS and similar insurance systems is to deliver cost-effective outcomes for tax payers.

A case in point is the statin group of cholesterol-lowering drugs, considered interchangeable for PBS reference pricing purposes. In August 2005, generic brands of simvastatin (principal brand name Zocor) were listed on the PBS for the first time, and a reduction in the price paid by the government was to affect all statin products (as a consequence of the 12.5% mandatory price cut in such circumstances), including Pfizer's still-patented Lipitor. A special submission by Pfizer to the Pharmaceutical Benefits Advisory Committee (PBAC) was required for Lipitor to retain its price 'on the grounds that Lipitor is more effective than simvastatin in lowering cholesterol' [9]. Clearly, the brand industry would rather not have to engage in such battles whenever regulators seek to reference a patented drug against off-patent alternatives. The present system makes this eventuality increasingly likely, as more patents expire and the 'innovator industry' continues to 'churn out' modified versions of existing drugs (with new patents) and 'me-too' products bringing marginal (if any) therapeutic benefits, rather than break-through products opening up new 'blockbuster' (billion dollar plus) markets.

The central role of Medicines Australia in the formation of the new generics policy may at first glance seem surprising since it is likely to result in an accelerated loss of market share for originator products. But the major gain for its member companies is a weakening of the reference pricing system, which will impact on the way that cost-assessment analyses are undertaken as part of the PBS listing process, through separation of all PBS items into two groups, F1 and F2, subject to different pricing arrangements. F1 medicines are those for which only a single brand is listed, mainly because of a patent, but will presumably also include off-patent drugs with only one supplier. F2 medicines have direct competition and are divided into two groups: one for products 'where price competition between brands is low' and one 'where competition between brands is high', i.e. those for which large discounts have been provided. Prices for products in the first group will be cut by 2% over three years from 2008, and those in the latter group will be subject to a one-off price drop of 25% on 1 August 2008. Price disclosure, that is, a requirement that the actual price paid by pharmacists be made known to the government, will be introduced over time for drugs in both groups. Pharmacists will be compensated for the impact of these adjustments in the period of the current Government-Pharmacy Guild agreement (which runs until mid-2010) through increases in the dispensing fee and the mark-up, and some other new incentive payments [10].

It is too early to assess the implications of these changes. Many aspects of the new arrangements are yet to be finalised, such as how price disclosures are to be administered. But a likely effect will be higher prices for newly listed (non-breakthrough) patented drugs than would have been the case under the present model. The details of the legislation, which is expected in the present term of parliament, are to be worked through within a small group of individuals from industry and government, apparently excluding consumer representatives.

A further reason for Medicines Australia not to be overly troubled by these changes is that its member companies are the suppliers, directly or indirectly, of many generics. When launching the new policy, Minister Abbott asserted that '70 percent of the Australian generics market is occupied by companies which are not members of the Generics Medicines industry Association' (GMiA) but members of Medicines Australia [2]. Indeed, in Australia and internationally, the historical dividing line between the generics and originator sectors has been much weakened, a development explored in my article on 'The global biopharma industry and the rise of Indian drug multinationals'. This has been made evident by, for example, the role of Novartis as the world's second largest generics supplier, through its subsidiary Sandoz. Sanofi-aventis has a similar subsidiary, while Alphapharm and other generics companies also market patented products. Originator companies also manipulate markets through the shadowy practice of 'authorised generics', launched to forestall genuine competition, common not only in the US but also in Australia. These are originator products marketed by, or licensed from, the originator company but sold under a generic name [11-13]. Faunce and Lexchin argue that 'linkage evergreening' may loom as an increasing threat to a sustainable generics industry in Australia, and argue for the public health benefits of a regulatory agency to police this area similar to Canada's Office of Patented Medicines and Liaison.

For their part, pharmacists are pleased that they will be largely compensated for the projected loss of income from the winding back of discounts. Changes to pharmacy remuneration will, according to the Health Minister, 'ensure that the position of pharmacists for the life of the current [government-PGA] agreement is as it would otherwise have been' [2]. Most consumers will experience no direct effects though it is possible that 'general consumers' paying the full co-payment ($30.70 in 2007) will pay less for some off-patent drugs priced below this level.

The argument pressed by Medicines Australia, and accepted by other stakeholders, is that lower generics prices will free up financial resources for the listing of new patented medicines and allow for PBS prices that better reflect 'rewards for innovation', including 'incremental innovation', that is, marginal improvements that regulators may consider to be of little if any therapeutic benefit. The pivotal claim by 'big pharma' companies, supported by the US government in trade negotiations and in other contexts, has long been that drug prices in Australia do not reward 'innovation' adequately [14,15]. Again, it is too early to assess the precise implications of the new policy, but the general point to be made in this context is that the cost-effectiveness principle – that the costs and incremental benefits of making available a new medicinal drug to a patient population should be compared with those of alternative treatments – applies logically across all segments of the pharmaceutical market, irrespective of how off-patent drugs are priced.

The new policy has not been welcomed by the GMiA which believes that it will weaken incentives for the use of 'true generics' [16]. The likely business dynamics of the generics sector in the new policy environment is indeed uncertain. Alphapharm and Sigma Pharmaceuticals presently control in excess of 80% of this market though Genepharm Australasia and other firms anticipate rapid expansion and growing market share on the assumption that present arrangements would not be radically altered. In response to the concerns of the GMiA, the Health Minister claims that the new system will provide 'systemic protection' for incumbent suppliers and 'should ensure that domestic generic manufacturers are less at risk from predatory newcomers such as some of the Indian generic drug manufacturers' [2]. The new arrangements are viewed in an analysis in the *Australian Financial Review *as 'carefully crafted to protect inefficient pharmacists and the local oligopoly of drug manufacturers'. The only opportunity for price competition which has been available to new generics entrants – discounts to pharmacists – is now being removed 'and the local manufacturers will be breathing easier' [17]. This points to the unanswered question of what form competition will take in the generics market as price disclosure is phased in. But there is no doubt that international and in particular Indian low cost suppliers such as Ranbaxy, which now has a presence in Australia, in coming years will have a significant impact, in one way or another, on the PBS market.

## Competing interests

The author(s) declare that they have no competing interests.
